# Exercise Physiology and Pulmonary Hemodynamic Abnormality in PH Patients with Exercise Induced Venous-To-Systemic Shunt

**DOI:** 10.1371/journal.pone.0121690

**Published:** 2015-04-28

**Authors:** Jian Guo, Xue Shi, Wenlan Yang, Sugang Gong, Qinhua Zhao, Lan Wang, Jing He, Xiaofang Shi, Xingguo Sun, Jinming Liu

**Affiliations:** 1 Department of Pulmonary Function Test, Shanghai Pulmonary Hospital, Tongji University School of Medicine, Shanghai, 200433, China; 2 Department of Pulmonary Circulation, Shanghai Pulmonary Hospital, Tongji University School of Medicine, Shanghai, 200433, China; 3 State Key Laboratory of Cardiovascular Disease, Heart-Lung Function Testing Center, Fuwai Hospital, National Center for Cardiovascular Diseases, Chinese Academy of Medical Sciences and Peking Union Medical College, Beijing, 100037, China; Virginia Commonwealth University, UNITED STATES

## Abstract

**Objectives:**

To identify the pulmonary hypertension (PH) patients who develop an exercise induced venous-to-systemic shunt (EIS) by performing the cardiopulmonary exercise test (CPET), analyse the changes of CPET measurements during exercise and compare the exercise physiology and resting pulmonary hemodynamics between shunt-PH and no-shunt-PH patients.

**Methods:**

Retrospectively, resting pulmonary function test (PFT), right heart catheterization (RHC), and CPET for clinical evaluation of 104 PH patients were studied.

**Results:**

Considering all 104 PH patients by three investigators, 37 were early EIS+, 61 were EIS-, 3 were late EIS+, and 3 others were placed in the discordant group. PeakVO_2_, AT and OUES were all reduced in the shunt-PH patients compared with the no-shunt-PH subjects, whereas VE/VCO_2_ slope and the lowest VE/VCO_2_ increased. Besides, the changes and the response characteristics of the key CPET parameters at the beginning of exercise in the shunt group were notably different from those of the no shunt one. At cardiac catheterization, the shunt patients had significantly increased mean pulmonary artery pressure (mPAP), mean right atrial pressure (mRAP) and pulmonary vascular resistance (PVR), reduced cardiac output (CO) and cardiac index (CI) compared with the no shunt ones (P<0.05). Resting CO was significantly correlated with exercise parameters of AT (r = 0.527, P<0.001), OUES (r = 0.410, P<0.001) and Peak VO_2_ (r = 0.405, P<0.001). PVR was significantly, but weakly, correlated with the above mentioned CPET parameters.

**In Conclusions:**

CPET may allow a non-invasive method for detecting an EIS and assessing the severity of the disease in PH patients.

## Introduction

Pulmonary hypertension (PH) is a progressive and usually life-threatening disease [1–3]. In this population, we frequently observed gas exchange patterns indicating acute hyperventilation caused by the venous-to-systemic shunting through a patent foramen ovale (PFO) directly into the systemic circulation [4–5], which regularly occur at the onset of clinical cardiopulmonary exercise testing (CPET).

PFO is found in nearly 30% of the adult population. The foramen ovale’s functional closure occurs postnatally as left atrial pressure exceeds that in the right atrium [6]. However, the foramen ovale does not spontaneously seal in a substantial number of persons [7]. Whenever right atrial pressure exceeds left atrial pressure, blood may shunt through the potential interatrial channel [7]. With regard to the patients with PH, as pulmonary vasculopathy progresses, pulmonary arterial resistance goes up during exercise as flow mediated dilatation of the lung circulation cannot increase appropriately. Eventually, venous return can shunt through a PFO to the systemic circulation, stimulating systemic arterial chemoreceptors and causing hyperventilation of the unshunted pulmonary blood flow as sun et al explained in the previous study [5].

Although CPET with gas exchange measurements has proved to be a powerful tool to grade the severity of exercise limitation, to detect exercise-induced venous-to-systemic shunting (EIS), to assess responses to therapy, and to predict prognosis in PH patients [8–10], there is little information in the current literature on the difference of exercise capacity and ventilatory effciency between shunt-PH and no-shunt-PH patients. Based on the aforementioned situation, our purpose was to identify the PH patients who develop an EIS using CPET and compare the exercise physiology and resting pulmonary hemodynamic between shunt-PH and no-shunt-PH patients.

## Materials and Methods

The medical records of 104 patients with idiopathic/heritable pulmonary arterial hypertension (I/HPAH) and chronic thromboembolic pulmonary hypertension (CTEPH) who systematically underwent resting pulmonary function test (PFT), CT angiography, right heart catheterization (RHC), and CPET for clinical evaluation in Shanghai Pulmonary Hospital between 2010 and 2013 were retrospectively studied. All of the participants that were involved in the study signed written informed consent. This study was approved by the Institution of Human Subjects Committee at Shanghai Pulmonary Hospital. The diagnosis of PH was based on clinical and laboratory data, which included RHC to satisfy diagnostic criteria according to currently accepted diagnostic criteria (Nice, 2013) [11]. RHC was performed within one month of each patient’s PFT and CPET studies. Patients had CPET just after their PFT studies. For comparison purposes, we analyzed the CPET and PFT data of 35 healthy subjects of similar age, gender, and body size. All PH patients also performed 6-minute walk testing (6MWT) according to the standards of the American Thoracic Society [12] and their blood samples were collected to detect n-terminal natriuretic peptide type-B (NT-proBNP).

### Resting pulmonary function measurements

Each subject underwent resting PFT of forced vital capacity (FVC), forced expiratory volume in 1 second (FEV_1_), diffusing capacity for carbon monoxide (DL_CO_), residual volume (RV) and total lung capacity (TLC) using standard methodology [13] and equipment (Jaeger Corp., Hoechberg, Germany). All resting lung function values were reported in absolute terms and normalized to percent of predicted (%pred). Predicted spirometry values were calculated using accepted equations for Chinese [14].

### Right heart catheterization

All patients underwent RHC. A True Size Thermodilution Catheter (Edward 774) was inserted via the left cubital. Mean hemodynamic measurements included mean pulmonary arterial pressure (mPAP), pulmonary artery wedge pressure (PAWP), and mean right atrium pressure (mRAP). Cardiac output (CO) was obtained using the thermodilution method. Pulmonary vascular resistance (PVR) was calculated using standard formulas: PVR = (mPAP-PAWP)/CO.

### Cardiopulmonary exercise test measurements

All the patients and controls performed on a cycle ergometer using a breath-by-breath system according to the American Thoracic Society/American College of Chest Physicians Statement on CPET [15]. Before each test, the equipment was calibrated in accordance with manufacturer’s specifications using reference and calibration gases. Standard 12 lead electrocardiograms (ECGs) and pulse oximetry were continuously monitored. Arterial blood pressure (BP) was measured every two minutes with an automatic cuff. The protocol was comprised of three minutes of rest, three minutes of unloaded cycling at 55–65 revolutions per minute (rpm), followed by a progressively increasing work rate of 5 to 15 watts (W)/min for PH patients and 20 to 25 W/min for the normal subjects to the maximum tolerance, and four minutes of recovery [16]. Direct measurements of oxygen uptake ($\dot{\text{V}}$O_2_), carbon dioxide output ($\dot{\text{V}}$CO_2_), minute ventilation ($\dot{\text{V}}$E), anaerobic threshod (AT), work rate (WR), end-tidal CO_2_ (PETCO_2_), end-tidal O_2_ (PETO_2_) and several derived parameters such as the heart rate (HR), oxygen pulse ($\dot{\text{V}}$O_2_/HR), respiratory exchange ratio (RER) and $\dot{\text{V}}$O_2_/$\dot{\text{V}}$E were obtained. Peak $\dot{\text{V}}$O_2_ was defined as the highest 30-second average of oxygen uptake in the last minute of exercise and other peak parameters were calculated at the same time. Each AT was determined by the V-slope method [17]. $\dot{\text{V}}$E-$\dot{\text{V}}$CO_2_ slope was determined by linear regression analysis of the relation between $\dot{\text{V}}$E and $\dot{\text{V}}$CO_2_ during exercise, excluding data above the ventilatory compensation point [18]. Lowest $\dot{\text{V}}$E/$\dot{\text{V}}$CO_2_ was determined by averaging the lowest consecutive 90 sec data points [18]. Thus OUES defines the slope of $\dot{\text{V}}$O_2_-vs-log$\dot{\text{V}}$E during an entire exercise period [19].

### Detection of EIS by gas exchange criteria

The presence of an EIS was determined by three investigators who were blinded regarding all the patients’ clinical course. 9-panel CPET plots were independently reviewed to identify an EIS during exercise using the following criteria as described in detail previously [5]: an abrupt and sustained increase in P_ET_O_2_, RER, $\dot{\text{V}}$E /$\dot{\text{V}}$O_2, $\dot{\text{V}}$_E /$\dot{\text{V}}$CO_2_ with a simultaneous, sustained decrease in P_ET_CO_2_ and pulse oximetry (SpO_2_) [5].

### Separation of PH patients into groups

Using above criteria, three investigators independently graded the 104 PH patients as either EIS positive (EIS+) or EIS negative (EIS-). Patients who were graded consistently as EIS+ by 3 graders were placed in the shunt group, those who were graded consistently as EIS- by 3 graders were placed in the no-shunt group and the others who were graded inconsistently were placed in the discordant group. During this process, three patients were identified as late-developing EIS just before the end of their CPET by all three graders independently and these patients were excluded from the grouping.

### Statistical analysis

Statistical analysis was performed using SPSS software (version 16, SPSS, Chicago). Parameters were expressed as mean±SD. Most PFT and CPET values are expressed in absolute terms and %pred. Unpaired Student t test was used to identify differences between groups, whereas χ2 test was used to assess differences in categorical variables between groups. Correlations between CPET parameters and hemodynamic variables were determined by Pearson’s correlation test. P value of < 0.05 was considered significant.

## Results

### Baseline clinical characteristics

The characteristics of PH patients and the controls were summarized in Table 1. The female-to-male ratio of the PH patients and healthy subjects in this study was about 2:1. Sex, age, height, weight, or BMI of the shunt, no-shunt, and control groups were similar (Table 1). The PFT parameters (FVC, FEV1, MVV, DL_CO_) were similar in the 2 PH patient groups, but dissimilar from those in the control group (Table 1). However, six minute walk distance (6WMD) was significantly lower in shunt PH patients than the no shunt ones. (394±99m versus 453±100m, p<0.05) (Table 1)

**Table 1:** Demographics, 6MWD, Pulmonary Function Testing and Cardiopulmonary Exercise Testing Parameters in PH Patients and Control Subjects.

Parameters	Shunt-PH Patients (n = 37)	No Shunt-PH Patients (n = 61)	Control Subject (n = 35)
**Age, yrs**	39±10	43±16	44±15
**Sex, female/male**	23/14	36/25	24/11
**Height, cm**	161±7	161±10	160±9
**Weight, kg**	57±9	58±9	56±8
**NYHA class**	2.9±0.6	2.7±0.5	—
**FVC, l(%pred)**	2.8±0.7(80.8±16.9)	2.8±0.9(82.7±18.9)	3.4±0.7(101.5±11.2)#
**FEV1, l(%pred)**	2.2±0.6(74.4±17.9)	2.1±0.7(74.8±19.7)	2.6±0.5(98.5±11.3)#
**FEV1/FVC,%**	74.2±8.4	74.9±13.2	77.6±4.5
**MVV, l/min(%pred)**	63.0±20.8(72.1±20.2)	67.3±30.1(72.4±28.8)	88.1±25.4(94.4±24.3)#
**DLco, ml/min/mmHg(%pred)**	16.8±5.2(82.1±18.8)	17.9±11.7(84.1±26.2)	22.7±5.0(110.5±17.2)#
**6MWD, m**	394±99*	453±100*	—
**Peak work rate, w(%pred)**	57.6±26.1(43.8±20.4)	66.2±24.8(51.8±22.0)	131.4±33.7(102.8±28.7)#
**Peak heart rate, beats/min**	138.7±20.1	135.3±25.2	158.1±15.6#
**Peak $\dot{\text{V}}$E, l/min**	43.8±12.2	44.2±14.6	60.5±14.4#
**Peak $\dot{\text{V}}$O2,ml/min (%pred)**	743.7±248.2(42.8±17.0)*	863.2±220.2(51.8±21.9)*	1609.2±346.9(71.4±17.6)#
**Peak $\dot{\text{V}}$O2/Kg, ml/min/Kg**	13.1±3.6*	14.8±3.7*	23.9±4.1#
**Peak O2 pulse, ml/beat**	5.3±1.3*	6.5±1.6*	10.8±2.3#
**AT, ml/min(%pred)**	542.5±134.3(47.7±11.5)*	649.5±170.5 (58.1±14.0)*	1000.1±232.4(94.8±19.0)#
**LOWEST $\dot{\text{V}}$E/$\dot{\text{V}}$CO2(%pred)**	55.2±12.3(206.2±41.8)*	47.9±10.7(176.8±38.2)*	30.0±2.9(107.1±10.0)#
**$\dot{\text{V}}$E/$\dot{\text{V}}$CO2 slope(%pred)**	70.0±26.2(242.1±87.9)*	50.3±16.6(174.4±60.7)*	26.2±2.7(94.1±10.0)#
**OUES, l/min/log(l/min) (%pred)**	0.9±0.3(50.9±25.0)*	1.2±0.3(75.3±37.6)*	2.0±0.4(140.7±53.5)#

Values are expressed as mean ± SD and percentage of measured to predicted values (%pred).

#p < 0.05, control group vs each group of PH patients;

*p < 0.05, shunt group versus no-shunt group using unpaired t test.

NYHA = New York Heart Association functional classification; FVC = forced vital capacity; FEV_1_ = forced expiratory volume in 1 second; MVV = maximum voluntary ventilation; DL_CO_ = gas transfer index or diffusing capacity for carbon monoxide; AT = anaerobic threshold; OUES = oxygen uptake efficiency slope; peak $\dot{\text{V}}$O_2_ = peak oxygen uptake, $\dot{\text{V}}$E = minute ventilation; $\dot{\text{V}}$CO_2_ = carbon dioxide output,

### Cardiopulmonary exercise analyses

**Fig 1:**
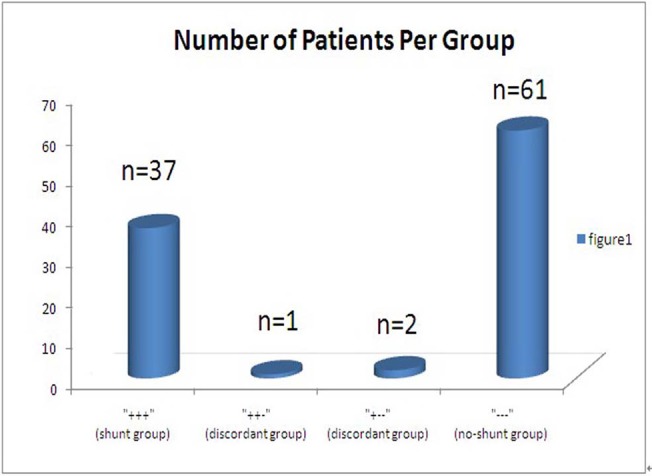
Performing a division of 101 PH patients into shunt, no-shunt and discordant groups. The columns quantify the decisions of three investigators blinded to each other independently. The presence or absence of an EIS during CPET is marked by—or +, respectively. All three graders described 61 patients as having no shunt and described 37 patients as having a shunt. In the two center columns, three patients are placed in discordant group because one of the investigators’ grades differed from the other 2.

All patients completed CPET without incident and exercised above their ATs. They all achieved a RER≥1.10, indicating sufficient metabolic stress. Excluding the 3 patients with a late-developing EIS, Fig 1 shows the distribution of the 101 patients among the no-shunt (n = 61), shunt (n = 37) groups (all 3 graders agreed) and the discordant (n = 3) group (one of the 3 investigators’ grades differed from the other two). The 3 discordant patients were excluded from considering. Thus, 40% ([37+3 = 40]/[104–3 = 101] = 40%) of our patients may have had EIS during exercise. Except for peak work rate, peak heart rate and peak ventilation, the usual peak exercise CPET parameters of the 2 PH groups were significantly dissimilar to each other (P<0.05). Peak $\dot{\text{V}}$O_2_, AT and OUES were all reduced in the shunt-PH patients compared with the no-shunt-PH subjects, whereas $\dot{\text{V}}$E/$\dot{\text{V}}$CO_2_ slope and the lowest $\dot{\text{V}}$E/$\dot{\text{V}}$CO_2_ increased, indicating that exercise capacity and ventilatory efficiency of the shunt-PH patients might be decreased (Table 1).

### Pulmonary hemodynamics

The pulmonary hemodynamic parameters of the two PH groups are shown in Table 2. The serum NT-proBNP concentration was significantly higher in shunt PH group than the no shunt one (641.34±536.59pg/ml versus 1141.33±959.5pg/ml, p<0.05). Besides that, at cardiac catheterization, the shunt patients had significantly increased mPAP, mRAP and PVR, reduced CO and cardiac index (CI) compared with the no-shunt ones (P<0.05) (Table 2).

**Table 2:** Hemodynamics in Shunt-PH and No Shunt-PH Patients.

	Shunt-PH(n = 37)	No Shunt-PH(n = 61)
**NT-proBNP, pg/mL**	1141.33±959.5*	641.34±536.59*
**mRAP, mmHg**	10.60±4.70*	7.62±4.56*
**mPAP, mmHg**	66.82±16.280*	58.49±19.413*
**CO, l/min**	4.01±1.32*	4.83±1.51*
**CI, l/min/m^2^**	2.46±0.55*	3.03±1.10*
**PVR, mmHg/l/min**	15.42±5.87*	11.57±7.31*
**PAWP, mmHg**	8.21±3.81	8.15±3.78

Values are expressed as mean ± SD.

*p < 0.05, shunt group versus no-shunt group using unpaired *t* test.

NT-proBNP = n-terminal natriuretic peptide type-B; mPAP = mean pulmonary artery pressure; mRAP = mean right atrial pressure; PVR = pulmonary vascular resistance; CO = cardiac output; CI = cardiac index; PAWP = pulmonary arterial wedge pressure

### Differences in gas exchange between shunt and no-shunt group

**Fig 2:**
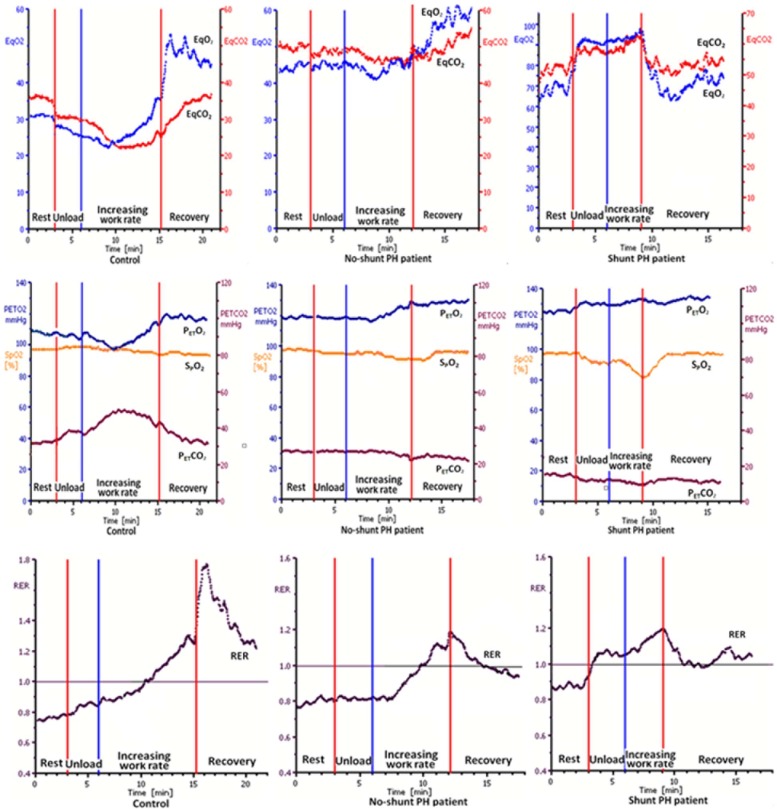
Differences in gas exchange of two representative PH patients (one shunt and one no shunt) and a normal subject. The responses of CPET in three female patients: one normal (aged 40 years; height, 160 cm; weight, 58 kg); one on-shunt-PH patient (aged 35 years; height, 157 cm; weight, 52 kg); and one shunt-patient (aged 38 years; height, 156 cm; weight, 54 kg). Symbols indicate 10-second averaged values. The course of CPET consisted of three minutes of rest, three minutes of unloaded cycling followed by increasing WR of 10 watts/min for PH patients and 20 watts/min for the control, and eventually four minutes of recovery. In the shunt-PH patient, PETO_2_, $\dot{\text{V}}$E/$\dot{\text{V}}$O_2_ (EqO_2_), $\dot{\text{V}}$E/$\dot{\text{V}}$CO_2_ (EqCO_2_) and RER concurrently abruptly increased while PETCO_2_ and SpO_2_ abruptly decreased. The no-shunt-PH patient showed less changes in PETCO_2_, PETO_2_, RER, $\dot{\text{V}}$E/$\dot{\text{V}}$O_2_ (EqO_2_), $\dot{\text{V}}$E/$\dot{\text{V}}$CO_2_ (EqCO_2_) and SpO_2_.

**Fig 3:**
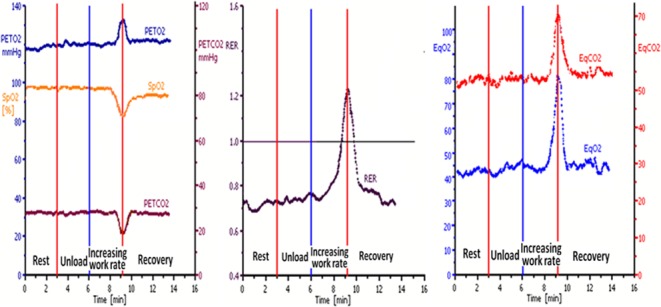
CPET of a PH patient who had a late-developing EIS at the end of exercise. The CPET course was comprised of three minutes of rest, three minutes of unloaded cycling followed by progressively increasing WR of 10 watts/min for the patient and eventually four minutes of recovery. Just before the end of exercise, the PH patient developed a late-developing EIS marked with the gas exchange findings of an abrupt increase in $\dot{\text{V}}$E/$\dot{\text{V}}$O_2_ (EqO_2_), $\dot{\text{V}}$E/$\dot{\text{V}}$CO_2_ (EqCO_2_), PETO_2_ and RER, accompanying with a simultaneous decrease in PETCO_2_ and SpO_2_, then these changes suddenly returned toward their pre-shunt values when exercise stopped.

Fig 2 shows the differences in gas exchange of 2 representative PH patients (one shunt and one no shunt) and a normal subject. Gas exchange of the PH patients was impaired (high $\dot{\text{V}}$E /$\dot{\text{V}}$O_2_,$\dot{\text{V}}$ E /$\dot{\text{V}}$CO_2_ and P_ET_O_2_ with low P_ET_CO_2_) at rest (Fig 2). After beginning the unloaded cycling, P_ET_O_2_, $\dot{\text{V}}$E /$\dot{\text{V}}$O_2_, $\dot{\text{V}}$E /$\dot{\text{V}}$CO_2_ and RER concurrently abruptly increased, while the P_ET_CO_2_ and SpO_2_ abruptly decreased in the shunt group. On the contrary, less changes were shown in P_ET_CO_2_, P_ET_O_2,_ RER, $\dot{\text{V}}$E /$\dot{\text{V}}$O_2_, $\dot{\text{V}}$E /$\dot{\text{V}}$CO_2_ in the no-shunt group. Fig 3 illustrates 1 of 3 patients who had a late-developing EIS just before stopping exercise.

Table 3 contrasts the changes in $\dot{\text{V}}$E, RER, P_ET_O_2_, P_ET_CO_2_, $\dot{\text{V}}$E /$\dot{\text{V}}$O_2_, $\dot{\text{V}}$E /$\dot{\text{V}}$CO_2_, and SpO_2_ from the rest to the ending of unloaded cycling that distinguish the no-shunt PH group from the shunt group.

**Table 3:** Changes in CPET Parameters from Rest to End of Unloaded Cycling in PH Patients and Control.

	Shunt-PH(n = 37)	No Shunt-PH(n = 61)	Control Subject(n = 35)
**Δ** $\dot{\text{V}}$ **E, l/min**	13.82±5.70* #	7.91±3.30* #	6.29±2.31#
**Δ** $\dot{\text{V}}$ **O_2_, l/min**	0.19±0.05	0.20±0.07	0.22±0.06
**Δ** $\dot{\text{V}}$ **CO_2_, l/min**	0.21±0.05	0.19±0.06	0.19±0.06
**ΔRER**	0.16±0.13* #	0.08±0.11*	0.04±0.06#
**ΔP_ET_O_2_, mmHg**	3.67±2.79* #	0.38±2.61* #	-3.02±3.43#
**ΔP_ET_CO_2_, mmHg**	-2.09±1.59* #	0.56±1.84* #	3.96±2.21#
**Δ** $\dot{\text{V}}$ **E /** $\dot{\text{V}}$ **O_2_**	11.59±15.88* #	-1.21±4.18* #	-3.98±3.90#
**Δ** $\dot{\text{V}}$ **E /** $\dot{\text{V}}$ **CO_2_**	1.59±16.66* #	-7.07±6.95*	-7.00±7.61#
**ΔSpO_2_,%**	-5.62±4.23* #	-1.21±1.32* #	-0.52±0.64#

Values are expressed as mean ± SD.

Δdenotes the changes from rest to the end of unloaded cycling exercise.

#p < 0.05, vs control group;

*p < 0.05, vs no-shunt PPH group using unpaired t test.

The abbreviation definitions are same as Table 1

### Correlations

Table 4 summarizes multiple correlations between CPET and hemodynamic variables for all the PH patients. Resting CO was significantly correlated with exercise parameters of AT (r = 0.527, P<0.001), OUES (r = 0.410, P<0.001) and Peak $\dot{\text{V}}$O_2_ (r = 0.405, P<0.001). PVR was significantly, but weakly, correlated with above mentioned CPET parameters. In contrast, neither of the hemodynamic variables correlated significantly with the PFT parameters for PH patients shown in Table 5, indicating that CPET parameters might be better to evaluate and predict the hemodynamic abnormality for patients with PH compared with the PFT variables.

**Table 4:** Correlation between Selected Resting Hemodynamics and Selected Exercise Parameters for PH Patients.

	AT	OUES	Peak$\dot{\text{V}}$O_2_
**CO**	0.527**	0.440**	0.421**
**PVR**	0.350*	0.401**	-0.330*

*:*p*<0.05;

**:*p*<0.001

The abbreviation definitions are same as Table 1 and Table 2.

**Table 5:** Correlation between Selected Resting Hemodynamics and Selected PFT Parameters for PH Patients.

	FVC	FEV_1_	RV	DL_CO_	Rtot
**CO**	0.133	0.067	0.230	0.140	-0.077
**PVR**	0.038	0.066	-0.210	-0.165	0.125

The abbreviation definitions are same as Table 1 and Table 2.

## Discussion

While prior studies have described the specific gas exchange changes that can be used to identify an EIS in IPAH patients from Harbor-UCLA [5], our study is the first to demonstrate the diagnostic utility of CPET in PH patients for an Asian population. In addition, our study compares the exercise physiology and resting pulmonary hemodynamics between shunt-PH and no-shunt-PH patients. In the present study, we showed that shunt-PH patients had impaired CPET responses compared with no-shunt-PH ones. Firstly, the usual CPET parameters of exercise capacity and gas exchange (Peak$\dot{\text{V}}$O_2_, AT and OUES) were all reduced in the shunt-PH patients compared with the no-shunt-PH subjects, whereas $\dot{\text{V}}$E/$\dot{\text{V}}$CO_2_ slope and the lowest $\dot{\text{V}}$E/$\dot{\text{V}}$CO_2_ increased (Table 1). Secondly, the changes and response characteristic of key CPET parameters at the beginning of exercise in the shunt group were notably different from those of the no-shunt ones. And thirdly, the shunt patients had significantly increased mPAP, mRAP, PVR and NT-proBNP, reduced CO, CI and 6MWD compared with the no-shunt ones. All of our results highly indicate that the patients with the presence of an EIS were more likely to have very severe PH.

Sun et al showed the high sensitivity and specificity in EIS detection comparing CPET with echocardiography and declared CPET is a safe, noninvasive, cost-effective, and easily repeatable method for detecting an EIS [5]. In our present study, we also find there is a big difference in the pattern of gas exchange that distinguishes the shunt from the no-shunt group. At the beginning of exercise, P_ET_O_2_, $\dot{\text{V}}$E/$\dot{\text{V}}$O_2_, $\dot{\text{V}}$E/$\dot{\text{V}}$CO_2_ and RER concurrently abruptly increased in the shunt group while P_ET_CO_2_ and SpO_2_ abruptly decreased, indicating an acute ventilatory increase disproportionate to metabolism [20–21]. Differently from that, the no-shunt PH patients showed less changes in P_ET_CO_2,_ P_ET_O_2_, RER, $\dot{\text{V}}$E/$\dot{\text{V}}$O_2_, $\dot{\text{V}}$E/$\dot{\text{V}}$CO_2_ and SpO_2_. Even at rest, the $\dot{\text{V}}$E/$\dot{\text{V}}$O_2_, $\dot{\text{V}}$E/$\dot{\text{V}}$CO_2_, and P_ET_O_2_ were higher and P_ET_CO_2_ was lower in the shunt than no-shunt group. These abnormalities could be attributed to hypoperfusion of well-ventilated lung and probable chronic hyperventilation as sun et al explained [5].

In our study, we also found four late-developing EIS near the end of exercise which were characterized by striking increases in P_ET_O_2_, RER, $\dot{\text{V}}$E/$\dot{\text{V}}$O_2_ and $\dot{\text{V}}$E/$\dot{\text{V}}$CO_2_, with concurrent abrupt and dramatic decreases in P_ET_CO_2_. The explanation to this finding could be that, at the end of exercise, the shunted blood is so acidemic and hypoxemic that it more strikingly stimulates ventilation to keep arterial homeostasis and the intolerable breathlessness forced the patients to stop [4–5].

In the present study, not only the pattern of gas exchange was different, but also the exercise capacity and ventilatory efficiency between shunt and no-shunt group was inconsistent. We noted that Peak$\dot{\text{V}}$O_2_, AT and OUES were all reduced in the shunt-PH patients compared with the no-shunt-PH subjects, whereas $\dot{\text{V}}$E/$\dot{\text{V}}$CO_2_ slope and the lowest $\dot{\text{V}}$E/$\dot{\text{V}}$CO_2_ increased. It is well-known that Peak$\dot{\text{V}}$O_2_, AT, $\dot{\text{V}}$E-$\dot{\text{V}}$CO_2_ slope, lowest $\dot{\text{V}}$E/$\dot{\text{V}}$CO_2_, and OUES are the most commonly used clinical parameters for diagnostic and prognostic information [8, 22]. A brief description of these CPET parameters that usually reflect exercise capacity and gas exchange in PH patients follows.

Peak$\dot{\text{V}}$O_2_ is frequently used as the most reliable measure of overall exercise capacity [23]. It is reduced in patients with higher PVR and lower CO and is highly correlated with the amount of functional pulmonary vascular bed [23]. The AT, which describes the highest $\dot{\text{V}}$O_2_ that the patient can sustain without developing a lactic acidosis, appears to be an independent marker of PH severity [21]. Recently, the values of $\dot{\text{V}}$E/$\dot{\text{V}}$CO_2_ during moderate exercise have been demonstrated as diagnostic and prognostic values in heart failure patients [24]. In PH patients, elevated $\dot{\text{V}}$E/$\dot{\text{V}}$CO_2_ levels manifest that the ventilation of underperfused alveoli causing an increase in dead space ventilation [21]. Increased $\dot{\text{V}}$E/$\dot{\text{V}}$CO_2_ levels have also been significantly correlated with decreased CO, elevated pulmonary arterial pressures, decreased alveolar-capillary membrane conductance, and diminished heart rate variability [25–27].

Oxygen uptake efficiency (OUE) represents the change in $\dot{\text{V}}$O_2_ as related to $\dot{\text{V}}$E. The most widely studied index of OUE is the oxygen uptake efficiency slope (OUES), which ordinarily mathematically defines the slope of $\dot{\text{V}}$O_2_-vs-log$\dot{\text{V}}$E during an entire exercise period [19]. OUES may have important prognostic value in exercise physiology in patients with chronic heart failure and was suggested as a submaximal index of cardiopulmonary functional reserve incorporating cardiovascular, musculoskeletal, and respiratory function [28–30].

The conclusion of older reports that severe PAH patients treated with atrial septostomy have reduced right atrial pressure, increased cardiac output and better survival does not conflict with ours [31–32]. An EIS is in fact a means of self-protection by decompressing the right ventricle. During exercise the shunt PH patients develop an EIS spontaneously to permit right ventricular unloading, that may be attributed to the especially high right ventricular pressure while the pressure in no shunt patients is not high enough. During cardiac catheterization in our study, the shunt patients had significantly increased mPAP, mRAP and PVR, reduced CO and CI compared with the no-shunt ones. The results noted above strongly indicate that patients with an EIS may have increased severity of disease than the no shunt patients. Our study also showed that several CPET parameters (AT, OUES and PeakVO_2_) were correlated significantly with the resting CO and PVR of the patients with PH, while neither of the hemodynamic variables correlated with the PFT parameters for PH patients. Although cardiac catheterization is essential in making the diagnosis, it is invasive with a big risk of mortality to PH patients [33–34]. However, CPET parameters used to detect aerobic function and ventilator efficiency might be better for estimating the severity of disease and following up the clinical course [35]. NT-proBNP and 6MWD are the very commonly used clinical parameters to predict diagnostic or prognostic information for patients with cardiac or pulmonary disease [12, 36]. In our study, the serum NT-proBNP concentration was significantly increased in the shunt PH patients compared with the no-shunt PH subjects, whereas 6MWD was reduced. Similarly, Oudiz et al also found that an exercise-induced venous-to-systemic shunt is highly predictive of poor outcome in patients with PH [8]. Although the exercise-induced shunting might let the patients get benefit from the ability to relieve pressure of their right ventricles, the venous-to-systemic shunt was more likely indicating the higher severity of pulmonary hypertension [8].

The drawback of our study is that the atrial venous-to-systemic shunt is not detected by resting echocardiography. Actually, several studies showed that during echocardiography the incidence of PFO in untreated PH patients was detected at just 5%-18% [37–39], while an autopsy study of nine hundred and sixty-five “normal” hearts showed the incidence of a PFO was 20% to 34% [7]. Sun et al found the incidence of venous-to-systemic shunting during exercise in IPAH patients was 38% to 45%. The explanation might be that shunting is a dynamic process and a PFO may be so tiny or the pressure differences of interatrial so trivial, echocardiography could not demonstrate the shunt blood flow [5]. In our PH patients, the incidence of venous-to-systemic shunting during exercise using CPET was about 40% which seems to be closer to the result of autopsy and sun’s study. In order to improve the accuracy of diagnosis in our study, 3 experienced investigators were arranged to independently reviewed the 9-panel CPET plots to identify an EIS. Only when the patients were graded consistently as EIS+ or EIS- by all 3 graders, were they placed in the shunt or no-shunt group.

## Conclusions

Our results from CPET highly indicated that the exercise capacity and ventilatory efficiency decreased in the shunt-PH compared with no-shunt-PH patients, and the key CPET parameters (AT,OUES and PeakVO_2_) were correlated significantly with the resting hemodynamic variables (CO and PVR). CPET may allow a non-invasive and relatively inexpensive method for detecting an EIS and assessing the severity of the disease in PH patients. Although it appears that patients with an exercise-induced shut have worse disease, further prospective studies evaluating its predictive value is warranted.
